# Menthol in the Treatment of Erysipelas

**Published:** 1891-05

**Authors:** A. L. Benedict

**Affiliations:** Buffalo, N. Y.


### MENTHOL IN THE LOCAL TREATMENT OF ERYSIPELAS.

By A. L. BENEDICT, M. D., Buffalo, N. Y.

G——, a man of middle age, had his first attack of erysipelas eighteen years ago, when the head and face were affected. Six years later the right foot was involved. In 1887 two attacks occurred in the left ankle and foot, there being a period of a week or two between the attacks. In the Spring of 1889, the right foot was again the seat of the disease with considerable lymphangitis along the course of the internal saphenous vein. This attack was treated locally with naphthalin ointment, and it lasted eight days from the initial chill to the defervescence, the highest temperature observed being 104.8 on the fifth day. Eight to ten days was the duration of all the attacks which the patient had experienced, and all of them were of moderate severity.

On February 10, 1891, at 10 A. M., the patient was seized with a chill. He walked home, about a mile, and went to bed. Ordinary domestic remedies and diffusible stimulants were employed but the chilly sensations did not disappear for nearly two hours. Beside the sensation of cold there was violent retching but without emesis. The temperature record is as follows:

| | |
|---|---|
| Feb. 10, 1 P. M. | 101.2 |
| Feb. 10, 7 " | 102.8 |
| Feb. 11, 9 A. M. | 102 |
| " " 4 P. M., acetanilid | 104 |
| " " 6 " " | 103.8 |
| " "10 " | 103 |
| Feb. 12, 9 A. M. | 101.2 |
| Feb. 12, 2 P. M. | 101.4 |
| Feb. 12, 10 P. M. | 101.1 |
| Feb. 13, 9 A. M. | 99 |
| Feb. 13, 10 P. M. | 99.8 |
| Feb. 14, 9 A. M. | 98.7 |

Partly on account of the writer's lack of faith in internal medication in erysipelas, partly on account of the chronic lithemic condition of the patient and the irritability of his stomach, the treatment was limited, with the exception of a few doses of acetanilid and some other symptomatic treatment, to the local application of a fifteen per cent. solution of menthol in liquid petrolatum. The writer has never heard of such a use of menthol; he was led to try it from its known physiological action and from some practical experience with it in the treatment of conditions of the respiratory apparatus. Most of the essential oils contain some principle which is anesthetic and antiseptic—actions which are manifestly called for in the local treatment of erysipelas and, therefore, it would not be surprising to learn that others have used menthol in this disease.

After the first thirty-six hours a marked diminution in the rash was noticed, corresponding to the decline of temperature. On February 14th, a small bleb was opened on the great toe, and on the next day no trace of disease remained, except the thickened skin of the foot and an ecchymosis on the great toe, bearing testimony to the original severity of the disease. On the evening of February 15th, the foot was washed with a warm solution of mercuric chloride (1:4000), and for several days the patient was convalescent. Late in the evening of February 20th, the patient had another chill, which proved to be the beginning of another attack of erysipelas, almost identical in its local lesion, temperature, and symptoms with that just described. The menthol treatment was resumed, and the attack had subsided by February 26th.

As far as this case is concerned, the theoretical considerations that led to the adoption of the menthol treatment were supported by the practical result. The patient had never had so speedy recovery under other treatment, and he declared that the usual discomfort and pain attending the attacks were almost entirely relieved by the menthol. The patient's family expressed a decided preference for the odor of menthol as compared with that of naphthalin. The second attack—and that it was a second attack and not a recrudescence is plain when we consider the five days of normal temperature and the lack of either local or constitutional symptoms—is easily explained. Owing to a disturbance in the domestic economy, the patient's room was at the time the only one in which he could be made comfortable, and thorough disinfection of the room while he was in it was impossible. In some way, perhaps on account of the tenderness of the skin of the foot, possibly—though the patient will not admit this—through some minute abrasion, produced by picking off the dead cuticle, germs entered from the air of the room and produced the second attack.

The writer does not wish to be included among that deplorably large class of physicians who rush to brilliant generalization from one or two apparently satisfactory results. One swallow does not make a summer, and one case does not establish the efficacy of a drug. This case is reported for the sake of inviting others to add their testimony, and, according to the favorable or unfavorable evidence of a considerable number of cases, let the new treatment be judged.

